# Association between craniofacial patterns and third molar agenesis in orthodontic patients

**DOI:** 10.1007/s00056-023-00484-0

**Published:** 2023-06-28

**Authors:** Eva Paddenberg, Alice Correa Silva-Souza, Ariane Beatriz Blancato, César Penazzo Lepri, Peter Proff, Erika Calvano Küchler, Christian Kirschneck

**Affiliations:** 1 Department of Orthodontics, University of Regensburg, Regensburg, Germany; 2 Department of Restorative Dentistry, School of Dentistry of Ribeirão Preto, USP-University of São Paulo, Ribeirão Preto, SP Brazil; 3 Department of Biomaterials, University of Uberaba, Uberaba, Brazil

**Keywords:** Tooth agenesis, Malocclusion, Cephalograms, Panoramic radiography, Skeletal class, Zahnaplasie, Malokklusion, Kephalogramme, Orthopantomogramm, Skelettklasse

## Abstract

**Purpose:**

Third molar agenesis (TMA) is the most common craniofacial anomaly and has been associated with craniofacial patterns in different populations. Therefore, the aim of this retrospective cross-sectional study was to assess a possible association between craniofacial patterns and TMA in German orthodontic patients.

**Methods:**

Patients undergoing orthodontic treatment with dental records including anamnesis, pretreatment lateral cephalograms and orthopantomograms were evaluated. Cephalometric analyses were conducted digitally and lines, angles and proportions were measured to investigate craniofacial morphology. Skeletal classes were determined by the individualised Wits appraisal and ANB angle. The TMA was identified with the help of orthopantomograms. Patients showing agenesis of at least one third molar were included in the TMA group. Statistical analysis was performed to assess the association between TMA and craniofacial patterns (α of *p* ≤ 0.05).

**Results:**

A total of 148 patients were included, 40 (27.0%) presented at least one missing tooth (TMA group) and 108 (73.0%) showed full dentition (control group). Skeletal class determined by the individualised Wits appraisal revealed statistical significance between the TMA and control groups (*p* = 0.022), in which TMA patients were 11 times more likely to present with an individualised skeletal class III (odds ratio 11.3, 95% confidence interval 1.7–139.5). Skeletal cephalometric analysis revealed no statistical differences between TMA and control groups for any further angular, linear and proportional parameters.

**Conclusion:**

Third molar agenesis was associated with skeletal class III determined by the individualised Wits appraisal.

## Abstract (de)

**Ziel:**

Die Aplasie des dritten Molaren (TMA) ist die häufigste kraniofaziale Anomalie und wurde in unterschiedlichen Kollektiven mit kraniofazialen Mustern in Verbindung gebracht. Ziel dieser retrospektiven Querschnittsstudie war es daher, einen möglichen Zusammenhang zwischen kraniofazialen Mustern und TMA bei deutschen kieferorthopädischen Patienten zu untersuchen.

**Methoden:**

Ausgewertet wurden Patienten, die sich einer kieferorthopädischen Behandlung unterzogen und deren zahnärztliche Unterlagen vorlagen, einschließlich Anamnese, seitlicher Kephalogramme vor der Behandlung und Orthopantomogramme. Die kephalometrischen Analysen wurden digital durchgeführt, und es wurden Linien, Winkel und Proportionen gemessen, um die kraniofaziale Morphologie zu untersuchen. Die Skelettklassen wurden anhand der individualisierten Wits-Beurteilung und des ANB-Winkels bestimmt. Die TMA wurde mit Hilfe von Orthopantomogrammen identifiziert. Patienten, die eine Aplasie von mindestens einem dritten Molaren aufwiesen, wurden in die TMA-Gruppe aufgenommen. Die statistische Analyse wurde durchgeführt, um den Zusammenhang zwischen TMA und kraniofazialen Mustern zu bewerten (α von *p* ≤ 0,05).

**Ergebnisse:**

Insgesamt wurden 148 Patienten eingeschlossen, von denen 40 (27,0%) mindestens einen fehlenden Zahn aufwiesen (TMA-Gruppe), während 108 (73,0%) ein vollständiges Gebiss hatten (Kontrollgruppe). Die durch die individualisierte Wits-Beurteilung ermittelte Skelettklasse ergab eine statistische Signifikanz zwischen der TMA- und der Kontrollgruppe (*p* = 0,022), wobei die TMA-Patienten 11-mal häufiger eine individualisierte skelettale Klasse III aufwiesen (Odds Ratio 11,3, 95%-Konfidenzintervall 1,7–139,5). Die kephalometrische Analyse des Skeletts ergab keine statistischen Unterschiede zwischen den TMA- und den Kontrollgruppen in Bezug auf weitere anguläre, lineare und proportionale Parameter.

**Schlussfolgerung:**

Eine Aplasie der dritten Molaren war mit der skelettalen Klasse III assoziiert, die durch die individualisierte Wits-Beurteilung bestimmt wurde.

## Introduction

Orthodontic diagnostics include radiographic image examination, comprising panoramic radiographs and lateral cephalograms, thereby allowing the detection of tooth agenesis and cephalometric analysis. Cephalometric analysis includes an evaluation of skeletal, dental and soft tissue parameters in the sagittal and vertical plane by comparing measured variables to norms. Craniofacial patterns such as skeletal classes I, II and III define the anteroposterior relation between the maxilla and mandible and can be determined among other measurements by the individualised ANB angle [22, 23].

Tooth agenesis is the congenital absence of one or more teeth. It can affect any primary or permanent teeth, but is more common in the permanent dentition and occurs most frequently in third molars [20] with a worldwide prevalence of 22.63% [1], although a wide range is reported in literature. Genetic and environmental factors affect tooth agenesis aetiology to a variable degree, leading to different frequencies among sexes, jaws and populations [1]. The association between tooth agenesis and specific craniofacial patterns has been observed in many studies and the results from these independent studies were pooled in a recent systematic review [28]. However, the existing results are heterogeneous, in which each study reported an association with different sagittal and/or vertical craniofacial measurements and patterns [2, 6, 7, 12, 14, 21, 24, 29]. Therefore, the aim of this retrospective cross-sectional study was to assess a possible association between craniofacial patterns, determined by lateral cephalograms and TMA in a group of German orthodontic patients.

## Materials and methods

### Sample

This research project was approved by the local Ethics Committee of the University of Regensburg (number 19-1549-101). Informed consent was taken from all patients and legal guardians. The assent was also obtained from any patient younger than 18 years at the time of the dental appointment.

For the presentation of this study, the STROBE (strengthening the reporting of observational studies in epidemiology) statement was followed as a guideline [34].

This retrospective cross-sectional study considered patients with German ancestors undergoing orthodontic treatment at the University Hospital of Regensburg or at private clinics in Regensburg, Germany, for eligibility. Dental records including anamnesis, pretreatment lateral cephalograms and pretreatment or later orthopantomograms (OPG) were evaluated. Therefore, a convenience sample of patients of any age or gender who had their pretreatment lateral cephalograms taken between January 2020 and April 2021 was screened. Sample size calculation was based on existing studies [2, 6, 7, 12, 21, 24, 29]. A significance level of 95% and a power of 80% were accepted for the purpose of sample size calculation, assuming a difference of 20% between the groups (ratio 2:1). The number of patients required was found to be at least 117 (39 cases and 78 controls).

To avoid distortion of the data, patients with syndromes, cleft lip and/or palate, other types of tooth agenesis, history of facial trauma or facial surgery, previous orthodontic treatment and cases where a previous extraction of third molar could not be confirmed were excluded from the study.

According to the presence or absence of third molar agenesis, the patients were allocated to the TMA or control group, respectively.

#### Cephalometric analysis

Due to the multicentric study design, the lateral cephalograms were taken with different devices and settings. The pretreatment lateral cephalograms of all included patients were imported as lossless TIF files into the software ivoris® analyze pro (Computer konkret AG, Falkenstein, Germany, version 8.2.15.110) and calibrated. Then, cephalometric analysis based on the technique of Segner and Hasund [30] was conducted digitally, where only skeletal parameters were considered for further analyses.

Lines, angles and proportions were measured to investigate craniofacial morphology [9, 25, 31, 32]. Skeletal class was determined by the individualised Wits appraisal and ANB angle, as reported in Paddenberg et al. [22] because this method, inspired by the methods of Järvinen [11] and Panagiotidis and Witt [23], takes skeletal variables into account, which significantly influence the individual Wits appraisal and ANB angle. Thereby, for each individual patient the deviation from an ideal norm value is calculated, which allows a more precise diagnosis of the true sagittal relationship between the maxilla and the mandible. To determine the skeletal class, the difference between the measured Wits appraisal according to Jacobson or the ANB angle according to Riedel and the corresponding individualised Wits appraisal or ANB angle was calculated using the regression formulae of Paddenberg et al. [22]: if this value was within the range of ±1°, skeletal class I was diagnosed, whereas a difference smaller than −1° resulted in skeletal class III and a difference larger than 1° in skeletal class II diagnosis.

Prior to the main investigation, 22 randomly selected cephalograms were analysed independently in duplicate and twice with a minimum time interval of 2 weeks to test inter- and intrarater reliability, respectively. Intraclass correlation coefficients (ICC) were used to calculate intra- and interexaminer reliability. Inter- and intraexaminer reliability showed significant and good agreement for both examiners (ICC range 0.91–0.97) as reported in Kirschneck et al. [15].

#### Diagnosis of third molar agenesis

TMA was identified through the assessment of pretreatment and treatment progress OPGs. All OPGs were examined using the same protocol and doing so, in all cases third molar agenesis became clearly apparent from the OPGs alone [15–17]. In case of doubt, more than one OPG of the patient was evaluated to confirm TMA diagnosis. Tooth agenesis was defined based on the age of the patients taking into account when initial third molar formation should be visible in the OPG [16, 17]. Each OPG was evaluated by one experienced dentist (ECK). To test intrarater reliability, 10% (*n* = 15) of randomly chosen OPGs were investigated twice with a 2-week interval. Kappa (κ) statistics indicated excellent intrarater reliability (κ of 1.0).

Patients showing agenesis of at least one third molar were included in the TMA group, whereas participants with all 32 permanent teeth present were allocated to the control group.

#### Statistical analysis

Statistical analysis was performed using SPSS® Statistics 28 (IBM, Armonk, NY, USA). According to the Kolmogorov–Smirnov test, Shapiro–Wilk test and visual inspection of Q‑Q diagrams, data were normally distributed. Therefore, to assess a possible association between TMA and craniofacial patterns, two-tailed t‑test and exact Fisher–Freeman–Halton test were conducted for metrical and categorical parameters, respectively. The significance level was set at *p* ≤ 0.05 for all analyses.

## Results

**Fig. 1:**
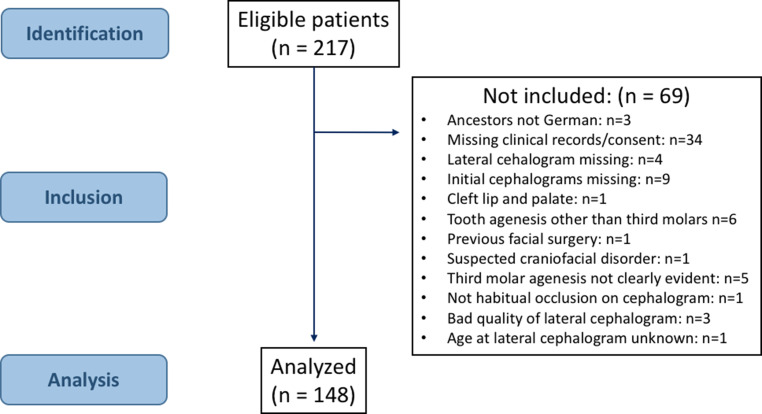
Patient’s flowchart Flussdiagramm zu den Patienten

Finally, 148 patients, aged between 7.3 and 36.1 years, were included in this study (Fig. 1).

Among the 148 evaluated patients, 40 (27.0%) presented at least one missing third molar (TMA group) and 108 (73.0%) showed all 32 permanent teeth developed (control group). Gender distribution was not statistically different among the TMA and control groups (χ^2^ test, *p* = 0.787) and (odds ratio 1.10, confidence interval 0.55–2.23).

The distribution of demographic characteristics among the groups and TMA characteristics are presented in Table 1. On average, TMA patients showed 2.3 missing third molars, and bilateral agenesis was more frequent (62.5%) than unilateral agenesis (37.5%).

**Table 1:** Demographic characteristics of the groups and TMA characteristics Demographische Merkmale der Gruppen und TMA-Merkmale

	Total sample (*n* = 148)	TMA (*n* = 40)	Control (*n* = 108)
*Age (years)*			
Range	7.3–36.1	9.0 to 27.4	7.3 to 36.1
Mean (SD)	12.6 (3.72)	13.7 (4.32)	12.3 (3.42)
*Gender (n, %)*			
Female	73, 49.3	19, 47.5	54, 50.0
Male	75, 50.7	21, 52.5	54, 50.0
*TMA characteristics (n, %)*^*a*^			
Maxillary agenesis	10, 25.0		
Mandibular agenesis	15, 37.5		
Both arches agenesis	15, 37.5		
Unilateral agenesis	15, 37.5		
Bilateral agenesis	25, 62.5		
Number of missing teeth, mean (SD)	2.3 (1.24)		

^a^% for TMA characteristics refers to a total of *n* = 40

*SD* standard deviation; *TMA* third molar agenesis; *%* relative frequency; *n* number of patients

The results of the skeletal malocclusions according to the groups are presented in Table 2. Skeletal class determined by the individualised Wits appraisal of Paddenberg et al. [22] revealed a statistically significant difference between the TMA and control groups (*p* = 0.022), in which TMA patients were 11 times more likely to present with an individualised skeletal class III (odds ratio 11.3, 95% confidence interval 1.7–139.5).

**Table 2:** Distribution of skeletal malocclusion according to the individualised Wits appraisal and ANB angle according to Paddenberg et al. [13] between the intervention (TMA) and control groups Verteilung der skelettalen Malokklusion entsprechend der individualisierten Wits-Beurteilung und des ANB-Winkels nach Paddenberg et al. [13] zwischen Interventions- (TMA) und Kontrollgruppe

Skeletal malocclusion^a^	TMA *n* (%)	Control *n* (%)	*p*-value
Skeletal class I, Wits	35 (87.5)	99 (91.7)	Reference
Skeletal class II, Wits	1 (2.5)	8 (7.4)	0.449^b^
Skeletal class III, Wits	4 (10.0)	*1 (0.9)*	*0.022*^b^*
Skeletal class I, ANB	14 (35.0)	39 (36.1)	Reference
Skeletal class II, ANB	22 (55.0)	61 (56.5)	0.999
Skeletal class III, ANB	4 (10.0)	8 (7.4)	0.723^b^

^a^According to Paddenberg et al. [13]

^b^ Exact Fisher–Freeman–Halton test was used instead of Pearson-Qui-squared^2^, because numbers were smaller than 5.

* significant at *p* < 0.05

*n* number of patients, *%* relative frequency, *TMA* third molar agenesis

Skeletal cephalometric analysis revealed no statistically significant differences between TMA and control groups for any further angular, linear and proportional parameters (Table 3).

**Table 3:** Angular, linear and ratio cephalometric measurement comparisons between TMA and control groups Vergleich der kephalometrischen angulären, linearen und Verhältnismessungen zwischen TMA- und Kontrollgruppen

Cephalometric parameter	TMA mean (SD)	Control mean (SD)	*p*-value^a^
*Angular measurements (°)*			
SNA	80.73 (3.94)	80.45 (3.60)	0.686
SNB	77.06 (3.39)	76.42 (3.21)	0.287
SNPg	78.05 (3.29)	77.47 (3.25)	0.337
ANB (Riedel)	3.67 (2.76)	4.04 (2.44)	0.430
NSBa	132.35 (5.01)	132.38 (4.92)	0.966
NSAr	125.56 (5.22)	125.77 (4.94)	0.828
ArGoMe	121.92 (6.73)	122.49 (6.73)	0.652
NGoAr	51.36 (4.01)	52.11 (3.94)	0.310
NGoMe	70.57 (5.04)	70.38 (4.35)	0.823
Compound angle	391.62 (5.54)	391.91 (5.61)	0.780
ML-NSL	31.59 (5.54)	31.84 (5.58)	0.806
NL-NSL	7.99 (4.01)	8.11 (3.17)	0.849
ML-NL	23.60 (5.39)	23.73 (5.75)	0.899
SN-Occl	18.46 (4.35)	19.27 (4.35)	0.314
Index (Hasund)	84.72 (7.40)	85.72 (6.23)	0.412
NSGn (y-axis)	67.79 (3.14)	67.92 (3.39)	0.838
Facial axis (Ricketts)	90.51 (3.26)	90.42 (3.55)	0.884
*Linear measurements (mm)*			
PgNB	1.58 (1.48)	1.76 (1.37)	0.481
Wits (Jacobson)	−0.48 (3.69)	0.08 (3.19)	0.364
*Ratio measurements (%)*			
SGo:NMe (Jarabak)	66.63 (4.78)	66.26 (4.83)	0.683

^a^ t-test for independent samples, two-tailed. Levene test showed variance homogeneity for all parameters at *p* ≥ 0.05

*TMA* third molar agenesis, *SD* standard deviation

## Discussion

The association between nonsyndromic tooth agenesis and other phenotypes such as craniofacial morphology [28], cancer [19], oral cleft [18] and other dental anomalies [16, 17] has aroused the interest of several research groups who have investigated different populations and outcomes worldwide. Tooth agenesis is characterized by a dental element that is congenitally missing. Clinically, a tooth germ fails to form between the age of its growth and development. The third molar, which is the most common missing tooth, develops entirely after birth and is the last tooth to erupt in all ethnic groups despite racial variations in the eruption sequence [12]. Our study included patients aged between 7 and 36 years. Jung and Cho [13] investigated third molar development in 2490 patients. The authors concluded that third molars appeared at the age of 6 years and had developed completely by the age of 24 years. Crypt formation was observed as early as at 7 years in the maxillary third molars and at 6 years in the mandibular third molars. The average age of the initial mineralization was 8.57 years [13]. Due to this late mineralization of the third molars, in some cases more than one OPG was evaluated. OPGs taken during or after orthodontic treatment were available and used for evaluation in younger patients. Therefore, patients were included in the study only when it was definitely possible to confirm the presence or absence of the tooth germ.

Several previous studies have reported the relationship between tooth agenesis and craniofacial morphology patterns [28], including smaller cranial base length [4], shorter maxilla [4, 33], skeletal class III [3] and a more prognathic mandible [4]. Some craniofacial patterns were associated with the most common type of tooth agenesis, which is TMA. Regarding the sagittal direction, several studies reported an association with TMA [11], e.g. an increasing prevalence of TMA was observed in cases with a smaller sagittal dimensions of the upper jaw [12], with reduced SNA or ANB angles [21] and with skeletal class III configurations [2]. In the vertical dimension, some authors did not observe an association with TMA [2]. However, other studies did show an association between TMA and certain vertical variables [6, 7], e.g. a meso- and brachyfacial pattern [6, 29] and a horizontal growth type [21, 24]. In our study, TMA was associated with skeletal class III if diagnosed according to the individualised Wits of Paddenberg et al. [22]. Celikoglu et al. [2] also observed that the percentage of TMA in skeletal class III subjects was higher than that in skeletal class I subjects in Turkish patients.

One important aspect to be highlighted here is that skeletal malocclusions were calculated as previously described in Paddenberg et al. [22]. Skeletal class can be determined with several cephalometric parameters, among which the ANB angle of Riedel [26] and the Wits appraisal of Jacobson [8] are widely well-known in orthodontic diagnostics. However, due to the variable configuration of the different craniofacial structures within an individual patient [5], these variables might not reflect the actual true sagittal discrepancy between the maxilla and the mandible. For example, the vertical orientation of the jaw bases influences the ANB angle [10, 23] and the inclination of the occlusal plane affects the Wits appraisal [11]. Thus, the ANB and Wits, which are directly measured, do not necessarily reflect the true anteroposterior relation of the maxilla and the mandible and empiric norm values do not allow a precise diagnosis of the sagittal relationship between the jaw bases. To consider the individual variation in the craniofacial pattern, individualised norm values should be used instead [30]. The advantage of the individualised norm values for the ANB and Wits appraisal, as described by Paddenberg et al. [22], is that several involved variables are included and that the regression coefficients used are up-to-date for a contemporary Central European population, which was analysed in our study.

Our results should be interpreted with caution. Different from previous studies [2, 6, 7, 11, 12, 21, 24, 29], linear, angular and ratio measurements were not associated with TMA. Other previous studies concluded that tooth agenesis had little [35] or no effect on craniofacial morphology [27]. A deep analysis of the studies [2, 6, 7, 11, 12, 21, 24, 29] leads to the observation that although there were statistically significant differences between tooth agenesis and full dentition groups, generally the mean values were within the normal range. Therefore, if there were small differences for the some of the measurements in our sample, these might only be detected in a larger sample size. Sample size was also a limitation of our study. Any stratified analysis of subgroups according to the affected arch was difficult because of only 10 cases in the maxillary and 15 cases in the mandibular arch presented with TMA.

Briefly, conducting cephalometric analysis by taking into consideration craniofacial pattern variables increases diagnostic precision. As our results indicate, the only skeletal cephalometric variable that showed an association between TMA and craniofacial patterns was the skeletal class determined by the individualised Wits appraisal, where a skeletal class III was more prevalent in the TMA group than in the control group. The cephalometric analysis applied here used individualised norm values, which seemed to be advantageous compared to empirical norms that solely reflect a population’s mean.

## Conclusions

Third molar agenesis was associated with skeletal class III, as determined by the individualised Wits appraisal.
